# Imaging Characterization of Renal Masses

**DOI:** 10.3390/medicina57010051

**Published:** 2021-01-08

**Authors:** Carlos Nicolau, Natalie Antunes, Blanca Paño, Carmen Sebastia

**Affiliations:** 1 Radiology Department, Hospital Clinic, University of Barcelona (UB), 08036 Barcelona, Spain; bpano@clinic.cat (B.P.); msebasti@clinic.cat (C.S.); 2 Radiology Department, Hospital de Santa Marta, 1169-024 Lisboa, Portugal; nhantunes@hotmail.com

**Keywords:** renal mass, renal cyst, renal cell carcinoma, ultrasound, computed tomography (CT), magnetic resonance imaging (MRI)

## Abstract

The detection of a renal mass is a relatively frequent occurrence in the daily practice of any Radiology Department. The diagnostic approaches depend on whether the lesion is cystic or solid. Cystic lesions can be managed using the Bosniak classification, while management of solid lesions depends on whether the lesion is well-defined or infiltrative. The approach to well-defined lesions focuses mainly on the differentiation between renal cancer and benign tumors such as angiomyolipoma (AML) and oncocytoma. Differential diagnosis of infiltrative lesions is wider, including primary and secondary malignancies and inflammatory disease, and knowledge of the patient history is essential. Radiologists may establish a possible differential diagnosis based on the imaging features of the renal masses and the clinical history. The aim of this review is to present the contribution of the different imaging techniques and image guided biopsies in the diagnostic management of cystic and solid renal lesions.

## 1. Introduction

The detection of renal masses is a common finding when using imaging techniques for different clinical purposes. Most of them are simple cysts that do not require further investigation, but complex cysts and solid masses are also common. Ultrasound (US), contrast-enhanced US (CEUS), computed tomography (CT), and magnetic resonance imaging (MRI) are the most common imaging techniques used to differentiate between benign and malignant lesions and to establish an appropriate management.

## 2. Imaging Tools to Characterize Renal Masses

Most renal masses are detected incidentally during a baseline US or a CT in the venous phase performed for a non-urological indication. The characterization of these masses (except for the typical simple cyst and fat-containing AML) requires a dedicated CT or MRI study after the administration of intravenous contrast agents. There is no consensus about the protocol for the characterization of renal masses using CT or MRI, but at least an unenhanced phase, a corticomedullary phase (25–70 s after contrast administration), and a venous phase (portal phase or preferably a nephrographic phase at approximately 100 s) are essential to determine the presence or absence of enhancement and to assess some features such as the vascularity [1] (hyper-, iso-, or hypo-enhancement) relative to the adjacent renal parenchyma, homogeneity, or heterogeneity of the enhancement and to determine more precisely whether it is an expansive or infiltrative lesion. Other phases like an excretory phase acquired 3 min after contrast administration are recommended by several authors. This can help in the assessment of the relation of the mass with the excretory tract and can help in establishing enhancing patterns of renal masses in different phases.

Regarding the choice of the optimal imaging technique for the characterization of renal masses, the diagnostic performance of CT and MRI is similar when based on the presence and type of enhancement. However, most guidelines recommend the preferential use of CT due to its greater availability, lower cost, better spatial resolution, and quality images without artifacts, and suggest using MRI for challenging cases, as is the case of the detection of a minimal amount of fat or when the lesion enhancement is equivocal [2]. However, the absence of ionizing radiation and the supplementary information provided by specific sequences such as diffusion imaging make MRI a more attractive and complete technique, and hence, depending on its availability, it may be considered the first diagnostic option. Moreover, the choice will depend not only on the initially performed test, but also on the experience of each center, with different complementary techniques, possible contraindications, and other patient characteristics. CEUS can also be used in several scenarios [3] with the advantage of real time evaluation, which allows a continuous assessment in all phases, with the additional advantages of lack of radiation and absence of nephrotoxicity of the US contrast agents.

With the continuous advances in imaging diagnosis, new tools are being incorporated such as CT texture analysis for the quantification of tumor heterogeneity [4], MRI diffusion and perfusion techniques [5,6,7], iodine quantification with dual-energy CT [8,9], and the incorporation of lesion segmentation software to determine with greater precision, for example, the degree of tumor enhancement [10]. However, further studies are necessary to know if these techniques are accurate and feasible enough to be incorporated in the routine imaging approach for characterization of renal masses.

## 3. Characterization of Cystic Masses

Renal cysts are the most common renal masses found incidentally when performing abdominal imaging examinations (US, CT, MRI) for different clinical purposes. A simple cyst is defined as a mass with fluid content with a thin (≤2 mm) and well-defined wall, without septa or soft-tissue nodules. Simple cysts present as thin-walled anechoic masses in conventional US, as hypodense (<20 HU), non-enhancing lesions in CT, and as structures hyperintense on T2-weighted and hypointense on T1-weighted images without abnormal contrast enhancement. Cystic masses that do not fulfill these criteria are defined as complex cysts usually secondary to the presence of intracystic hemorrhage or infection. Complex cysts should be characterized with the administration of contrast agent as up to 10% of renal cell carcinomas (RCCs) can present as complex cystic masses. The detection of enhancement in a cyst is not considered a sign of concern if detected in thin “hair-line” septa or wall. When using CEUS, the detection of microbubbles traveling in septa is a common finding that should not be interpreted as a sign of malignancy.

**Figure 1:**
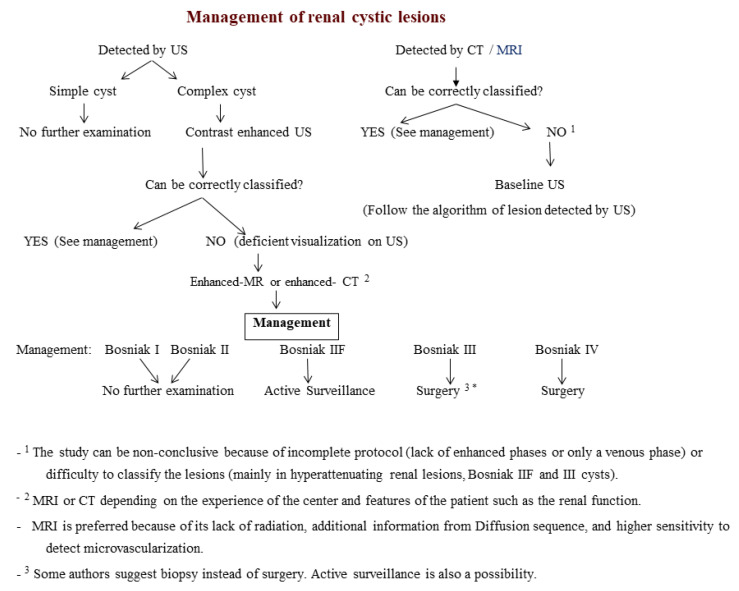
Diagnostic algorithm for the characterization of cystic renal masses. US: Ultrasound, CT: Computed Tomography, MRI: Magnetic Resonance Imaging.

A proposed algorithm for the diagnosis of cystic renal masses is summarized in Figure 1, and their classification is mainly based on the Bosniak classification.

A common situation is the detection of a homogeneous hyperattenuating renal lesion on CT. If the lesion shows an attenuation of 20 to 70 HU on unenhanced CT or >20 HU on single-phase enhanced CT, it is then considered indeterminate and further imaging investigation with dedicated CT, MRI, or US is required. However, characterization using US is particularly indicated for confirming the solid or cystic nature of a renal mass [11].

### Bosniak Classification

**Figure 2:**
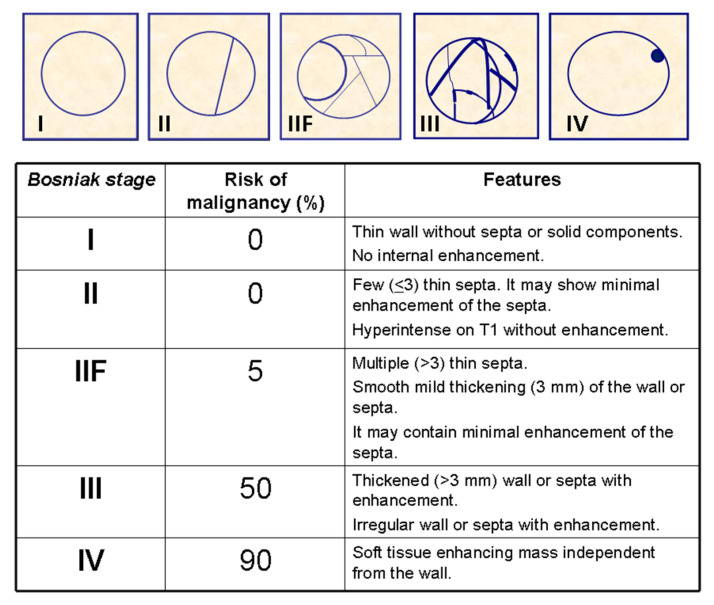
Main features of cystic renal masses following the Bosniak classification. Data in the table are adapted from previous and updated version [12].

The Bosniak classification was originally developed in 1986 to classify renal cysts based on CT findings, with a recent suggested update in 2019 [12]. It allows the classification of renal cysts when infection, inflammatory, or vascular etiologies have been ruled out based on different features. The administration of contrast agents is needed to evaluate the characteristics of the cystic masses [13,14] and the classification depends on features such as wall thickness; the presence, number, and thickness of septa; the attenuation or intensity of the lesion on CT or MRI, respectively; the presence of soft-tissue masses within the cyst; as well as the enhancement of the wall, septa, and intracystic soft-tissue masses (Figure 2).

The recent update of the Bosniak classification [12] makes an effort to clarify some undefined concepts of previous versions, specifying issues such as the number of thin septa (no more than three or more than three to differentiate between Bosniak II and IIF cysts); the differentiation between thin (≤2 mm), minimally thickened (3 mm), or thickened (≥4 mm) septa; and the differentiation between irregular thickening of the wall or septa (Bosniak III), and nodular thickening of the septa (Bosniak IV). However, this recent update will be difficult to implement as a difference of only 1 mm in the thickness of a septa can change the category of a complex cyst. In this update, cysts with smooth septa of 2 mm are considered Bosniak II, cysts with smooth septa of 3 mm are considered Bosniak IIF, and cysts with smooth septa of 4 mm are Bosniak III. Bosniak classification is predictive of the risk of malignancy and helps in the clinical management of cystic renal masses. Bosniak I and II cysts do not require further management, but Bosniak IIF cysts require follow-up due to the 5–10% risk of malignancy of theses cysts. A three-year follow-up is usually recommended, but some authors recommend five years due to the possibility of very slow growth of some cystic renal cancers [12,15]. A follow-up every 6 months the first year and then every year after is suggested. In contrast, surgery is recommended for Bosniak III and IV cysts due to a 50 and 90% probability, respectively, of being RCC. One of the most important limitations of the imaging management of complex cysts is the impossibility to differentiate between benign and malignant Bosniak III cysts [16] since both types of lesions show enhancing thickening of the wall and/or septa. However, in malignant cysts, the histologic examination will show neoplastic cells in the enhancing wall or septa. There is active work trying to develop a subclassification of Bosniak III lesions that will help to differentiate between less aggressive thickened septa and nodularity of the septa [17,18]. In addition, there is an intense debate about the management of Bosniak III lesions as 50% of these patients undergo surgery for benign lesions. Moreover, active surveillance (AS) would be a good option for patients with Bosniak III and IV cysts [19], mainly in those <2 cm or in patients at high-risk for surgery, comorbidities, or limited life expectancy, as cystic RCCs are usually low-grade and low-stage tumors with better prognosis than solid RCCs [20,21].

**Figure 3:**
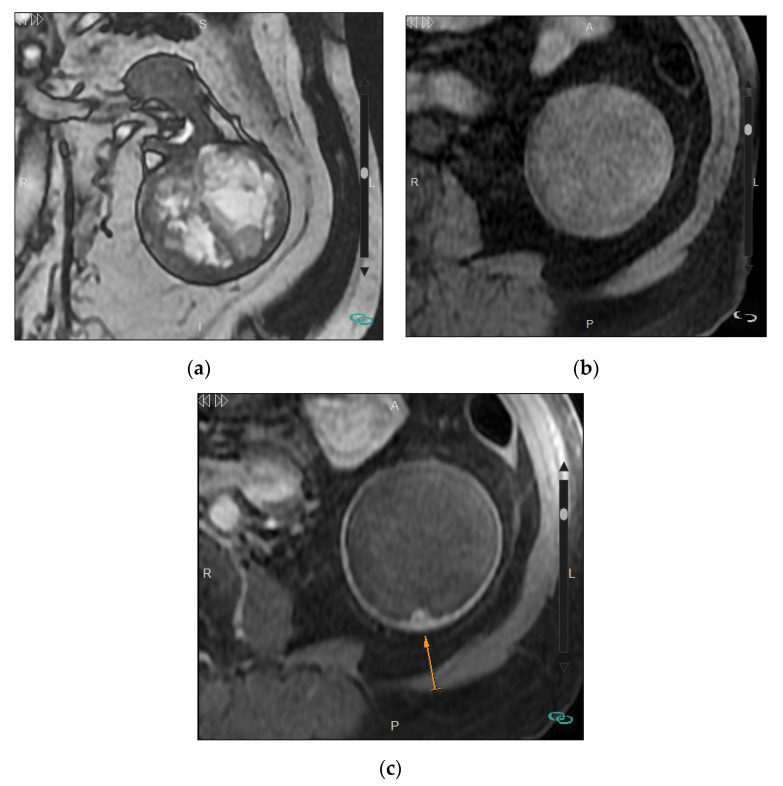
Bosniak IV cyst in a 77-year-old man. (**a**) Coronal fast spin-echo T2-weighted MRI shows a heterogeneous well-defined mass at the lower pole of the left kidney. (**b**) Pre-contrast T1-weighted image shows minimal heterogeneity of the mass. (**c**) Post-contrast T1-weighted image shows an 8 mm enhancing nodularity (arrow) arising from the posterior wall of a cystic mass corresponding to a Bosniak IV cyst.

**Figure 4:**
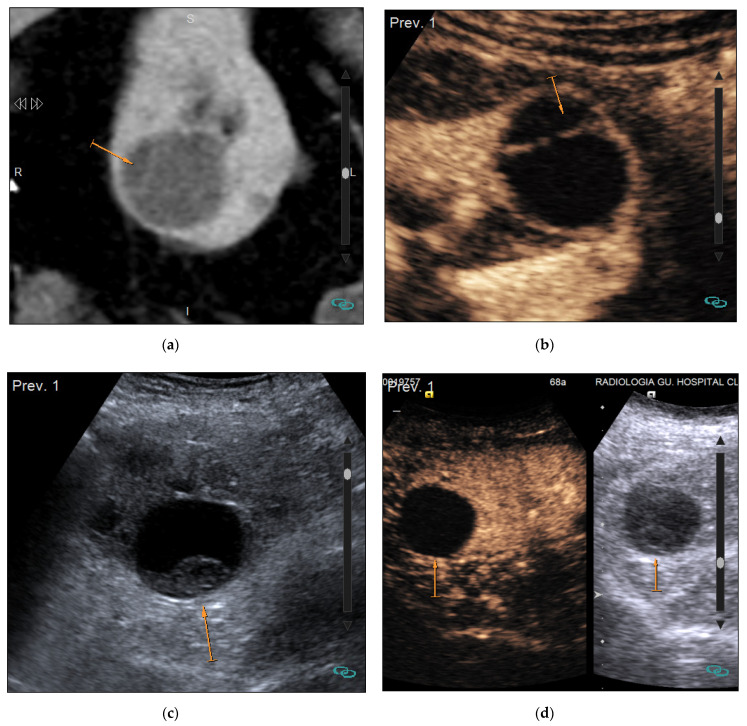
Evaluation of complex cysts with Contrast Enhanced Ultrasound (CEUS). (**a**) CT image shows a cyst with a thin septum (arrow) corresponding to a Bosniak II cyst in a 36-year-old woman; (**b**) CEUS image of the same cyst also shows the thin septum. Septa are usually better visualized by CEUS due to the possibility of real-time evaluation and its higher sensitivity for detecting microvascularization; (**c**) US image shows a complex cyst with suspicion of soft-tissue mass (arrow) in a 42-year-old woman; (**d**) CEUS image shows absence of enhancement of the intracystic lesion (arrow), corresponding to a Bosniak II cyst that does not require further examinations.

The Bosniak classification was originally developed to classify renal cysts based on CT findings, but MRI and CEUS can also be used [12,22,23,24] as the latter techniques show even higher sensitivity than CT for the detection of tumor microvascularization (Figure 3) [25]. This advantage and the absence of radiation make MRI and CEUS the recommended techniques not only for the characterization of complex cysts detected by US or CT, but also for follow-up when required and for characterization of CT indeterminate lesions [26,27]. With MRI, the use of substraction techniques can be useful to demonstrate the presence or absence of enhancement mainly in those lesions that show hypersignal on T1 sequences. In this setting, CEUS is very helpful in the detection of Bosniak I/II cysts that do not require further examination (Figure 4) and in the identification of papillary RCCs with very slight enhancement that may not be clearly detected on CT studies. One of the current controversies regarding the use of CEUS and MRI in the management of complex cysts is that they increase the detection of septa, thus, possibly increasing the detection of Bosniak III cysts that would have been characterized as Bosniak IIF by CT [28,29]. This increase of Bosniak IIIs lesion involves an increase in the number of surgical procedures that, as explained above, accounts for 50% of benign lesions.

Some authors recommend the use of other modalities such as MRI diffusion or biopsy [30,31,32]. In MRI diffusion, the presence of a thickened septum with restriction is suggestive of malignancy. A biopsy can also be helpful particularly in old patients, patients with one kidney, or patients at high-risk of surgical morbidity. However, there is not enough scientific evidence regarding the accuracy of both procedures in this setting.

## 4. Characterization of Solid Masses

When a solid renal mass is detected, CT and MRI are the recommended imaging modalities to characterize it, as US is not accurate enough. Small tumors are missed with US [33] and there is an overlap of the US features between benign and malignant lesions. Even the US features of small typical angiomyolipomas (well-defined homogeneous hyperechoic lesions) can be found in small RCCs [34]; thus, further examination (or follow-up in <10 mm lesions) is required to confirm the diagnosis. Color Doppler can help in the demonstration of intralesional flow, which would confirm that a lesion is vascularized. However, the absence of flow does not exclude malignancy as some malignant tumors such as papillary RCC can show minimal vascularity only detected after the administration of contrast agent. A simple way to classify solid masses is based on their shape. Nodular, well-defined (also called ball-type) renal masses are expansive lesions that result in a contour bulge. On the other hand, infiltrative, ill-defined (also called bean-type) renal masses grow while maintaining the renal shape. Different diagnostic possibilities are suggested depending on the shape of the renal masses.

### 4.1. Nodular Masses

**Figure 5:**
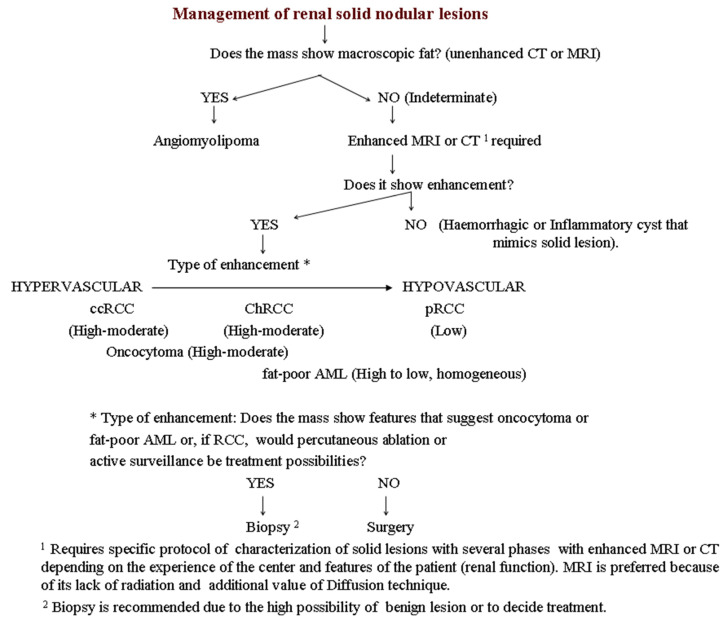
Diagnostic algorithm for the characterization of well-defined solid renal masses.

A proposed algorithm for the diagnosis of nodular renal masses is summarized in Figure 5.

RCC should be suspected when a nodular mass without macroscopic fat is detected. RCCs are the most common expansive solid masses, which, due to widespread use of imaging techniques, are frequently detected incidentally when they are small (<3 cm) and at an earlier stage, therefore having a better prognosis [35,36]. RCC encompasses a wide spectrum of histopathological entities with three main subtypes: clear cell carcinoma (ccRCC) accounting for 80% to 90% of all RCCs and have worse prognosis, and low-grade RCCs, which include papillary carcinoma (pRCC) and chromophobe carcinoma (chRCC), which account for 6–15% and 2–5%, respectively, with better survival rates [37,38]. In addition, pRCC is subclassified into type 1 (basophilic, usually low-grade) and type 2 (eosinophilic, usually high-grade), the latter with worse prognosis. The 2016 World Health Organization (WHO) classification of renal tumors also describes new renal subtypes that include multilocular cystic renal neoplasm of low malignant potential, MiT family translocation RCC, tubulocystic RCC, acquired cystic disease-associated RCC, clear cell papillary RCC, succinate dehydrogenase-deficient RCC, and hereditary leiomyomatosis and RCC-associated RCC [39].

**Figure 6:**
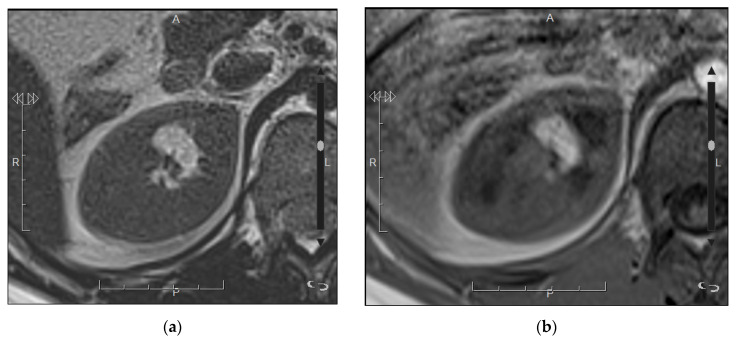

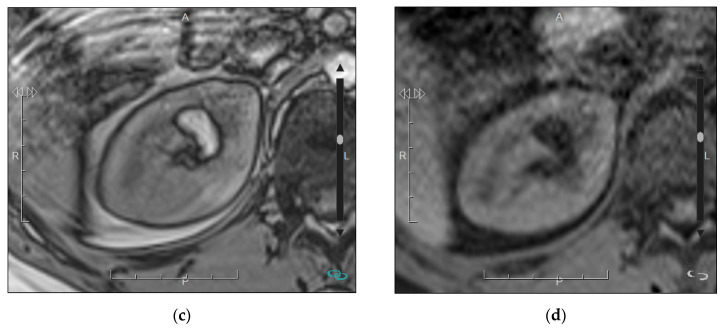
Angiomyolipoma in a 67-year-old woman. (**a**) Axial T2-weighted image shows a 2.3 cm hyperintense renal mass; (**b**) axial in-phase (**c**) and opposed-phase T1-weighted images show India ink artifact on the opposed-phase image; (**d**) axial fat-suppressed T1-weighted image shows low signal intensity of the mass. Scale size: 5 cm

When a solid renal mass is detected, the presence of macroscopic (extracellular) fat allows for the diagnosis of AML, as the presence of macroscopic fat in other renal tumors such as RCC is very uncommon and is usually associated with other features such as the presence of calcifications [2]. AMLs are tumors of mesenchymal origin composed of variable proportions of blood vessels, smooth muscle cells, and adipocytes [37]. They are highly prevalent in the general population (0.3–3%) and show two distinct epidemiological forms: the sporadic form (80% of cases, most common in middle age women) and those found in patients with tuberous sclerosis (20% of cases). The detection of macroscopic fat can be confirmed using CT (when the attenuation of the mass is <−20 HU) or using MRI (when the mass shows an iso-signal relative to the subcutaneous or intraabdominal fat in all phases, including a Fat-Saturation sequence) (Figure 6). It is important to notice that the detection of drop-signal (chemical-shift) in out-of-phase sequence indicates the presence of microscopic intracellular fat, and thus, it cannot be used for the diagnosis of AML. Although AML can contain microscopic (intracellular) fat, a high number of clear cell RCCs also contain fat [40,41].

If a well-defined solid mass does not contain macroscopic fat, the differential diagnosis includes mainly RCC, oncocytoma, and fat-poor AML. Other benign tumors such as renal adenoma or leiomyoma are rare, and other malignant tumors such as lymphoma and metastases unusually show a well-defined pattern (Table 1), making the clinical history, blood tests, and the presence of extrarenal findings helpful in the diagnosis. From a clinical point of view, it is important to differentiate between a surgical lesion and a non-surgical lesions (i.e., inflammatory pseudotumors, lymphoma, metastases). The most important tool to characterize solid lesions without obvious fat is the evaluation of the enhancement pattern after the administration of intravenous contrast agents (iodinated-based agents for CT and gadolinium-based agents for MRI). A remarkably high number of studies have evaluated the usefulness of different parameters such as the intensity, duration, and heterogeneity of the enhancement (including histograms and texture). Regarding the enhancement in different phases, ccRCCs are hypervascular and usually show hyperenhancement on the corticomedullary phase, whereas the papillary and chromophobe subtypes are less vascular and show their peak of enhancement at the nephrographic phase or even at the excretory phase in some very hypovascular pRCCs.

Regarding the detection of hypervascular lesions, the differentiation between ccRCC and oncocytoma should be considered. Oncocytomas are composed of oncocytes surrounded by thin capillaries and stroma and account for 3–7% of all solid renal masses, but their incidence increases to 18% in <4 cm tumors [37,42]. The mean age at presentation and male predominance are similar to RCC. Although most oncocytomas are unifocal, 2–12% are multifocal and 4–12% are bilateral [43,44]. Several associations have been described among patients with multifocal renal oncocytomas and hereditary syndromes such as familial oncocytosis and Birt–Hogg–Dubé syndrome [45,46]. Both ccRCCs and oncocytomas show similar enhancement [47,48], but the presence of a central scar and the inversion pattern of enhancement have been associated with oncocytomas [49,50]. However, a central scar is present in less than 50% of oncocytomas and few RCCs may show it [51,52]. On the other hand, the usefulness of the inversion pattern of enhancement (a hyperenhanced tumor segment on the corticomedullary phase reverts to hypoenhancing on the excretory phase) has not been demonstrated in several studies [53,54]. Other different CT features including lesion size (with lesions >4 cm more commonly corresponding to RCC), enhancement heterogeneity (more common in RCCs due to the presence of necrotic, cystic, or hemorrhagic areas), or a combination of features such as the difference between excretory enhancement and attenuation on unenhanced phase (significantly greater in oncocytomas than in RCCs) have been described as useful for the differential diagnosis [52,55,56,57,58]. However, there is still considerable overlap between ccRCC and oncocytoma regarding their enhancement features.

On the other hand, when homogeneous and prolonged enhancement is detected, the differentiation between low-grade RCCs and fat-poor AMLs should be considered. Fat-poor AMLs are lesions that contain less than 25% of fat on the histological evaluation [59,60], accounting for 4–5% of all AMLs. The presence of high attenuation on unenhanced CT is very suggestive of fat-poor AML [57]. Other features such as the presence of heterogeneity on CT texture analysis (more common in RCCs) [61], the arterial-to-delayed enhancement ratio (defined as the difference in attenuation between arterial and unenhanced phase divided by the difference between delayed and unenhanced phase, with values greater than 1.5 suggesting AML) [62], and the presence of low signal in T2 MRI images (commonly seen on AMLs) [59] can also be useful for the differential diagnosis. However, low-grade RCCs, especially the papillary type, may also show low signal on T2 sequence [59].

**Figure 7:**
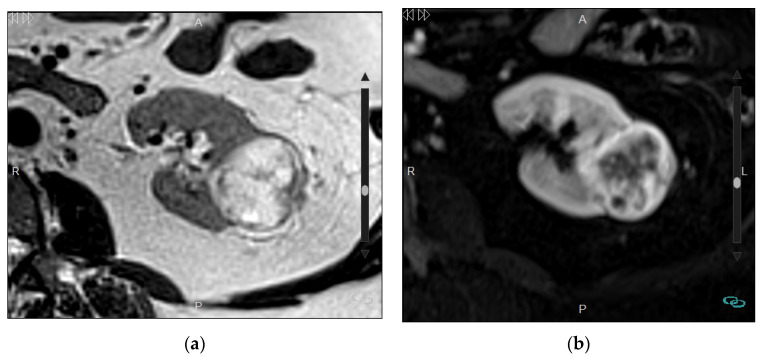

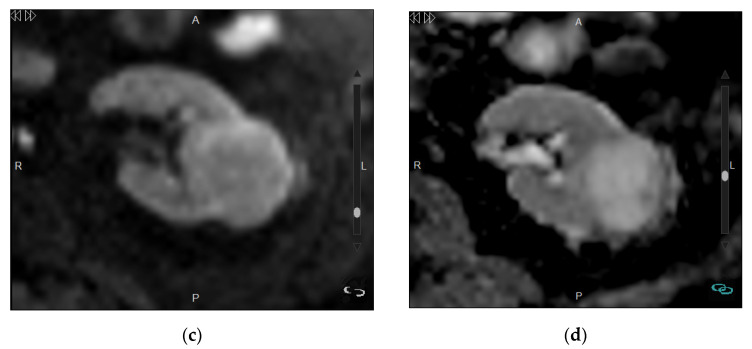
Oncocytoma in a 57-year-old man. (**a**) Axial T2-weighted MRI shows a nodular mass in the left kidney with heterogeneous but mainly hyperintense signal; (**b**) Axial contrast-enhanced T1-weighted image shows heterogeneous enhancement with intratumor hypoenhancing areas; (**c**) Axial diffusion-weighted image with high b = 1000 (**d**) and Apparent Diffusion Coefficient (ADC) map show absence of restriction.

**Figure 8:**
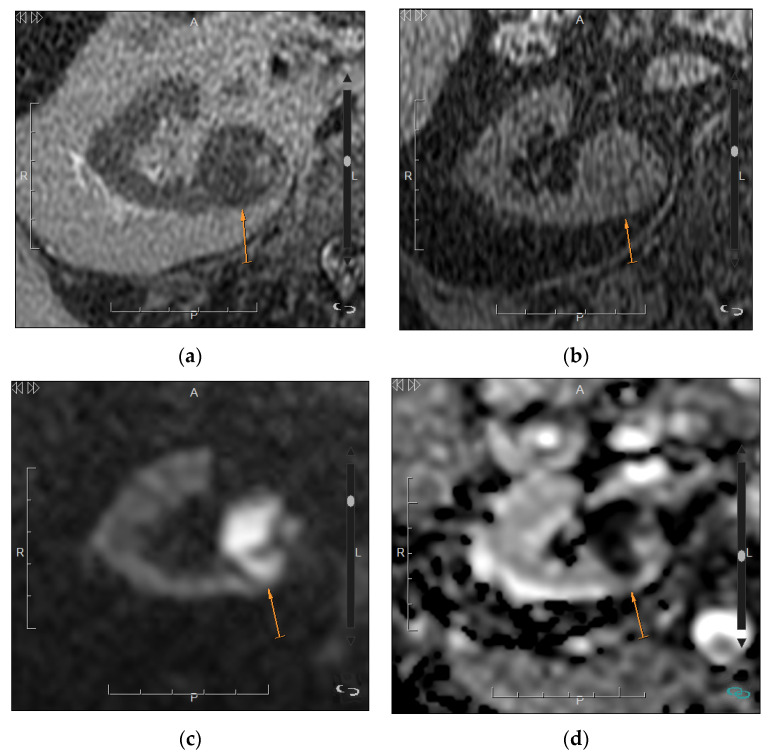
Papillary renal cancer in a 66-year-old man. (**a**) Axial T2-weighted MRI shows an almost complete low-signal mass (arrow) in a small kidney with a thin cortex due to chronic renal disease; (**b**) Axial fast spin-echo T1-weighted MRI shows isointensity of the mass without macroscopic fat (arrow); (**c**) Axial diffusion-weighted image with high b = 1000 (**d**) and ADC map show areas of restricted diffusion (arrow). No sequences after the administration of the contrast agent were performed due to the chronic kidney disease. Scale size: 5 cm.

Another imaging tool that can be helpful for the characterization of solid masses is diffusion weighted imaging (DWI). Recently, two meta-analysis have found significantly higher apparent diffusion coefficient (ADC) values in oncocytomas than in RCC (Figure 7) [7,63]. Other studies have also found significant differences in ADC between different subtypes of RCCs, with lower values in the papillary subtype (Figure 8) [64,65].

A summary of the imaging features of the most common solid nodular masses is shown in Table 2.

The accuracy of CT and MRI in the characterization of solid masses based on the morphology and enhancement patterns is similar. Most guidelines recommend the preferential use of CT for the characterization of renal masses due to its greater availability, lower cost, better spatial resolution, and quality images without artifacts, and suggest using MRI for inconclusive, challenging cases [2,62]. However, the absence of radiation and the supplementary information provided by the DWI sequence make MRI a more attractive and complete technique. In addition, the risk of nephrogenic systemic fibrosis associated with the use of gadolinium-based contrast-agents has dramatically decreased with the use of group II gadolinium-based contrast agents (gadobenate dimeglumine, gadobutrol, gadoterate meglumine, or gadoteridol). In a recent meta-analysis including 16 studies and 4931 patients, the use of group II gadolinium-based contrast agents in patients with stage 4 or 5 chronic kidney disease was associated with an extremely low risk of nephrogenic systemic fibrosis [66]. However, the clinical significance of another relevant issue, the possibility of gadolinium deposition in the brain, and in other tissues, remains unclear.

Due to the addressed limitations for the differential diagnosis of solid masses, there is growing support for their histological characterization using percutaneous biopsy. Most urological guidelines have incorporated biopsy in their diagnostic algorithms, not only to avoid surgery of benign lesions, but also to obtain more information regarding the type of RCC in order to decide the treatment among the available therapies (including the performance of biopsy before or during active surveillance, percutaneous ablation, or surgery) and to have accurate information about the prognosis in case of malignancy. Recent studies have shown the high accuracy of renal biopsies, with 90% in the study by Richard et al. [67] and 96% in the study by Maturen K [68]. Although there is no worldwide consensus regarding when to perform a biopsy in nodular solid lesions, the tendency is to biopsy lesions that cannot be fully characterized by imaging techniques, particularly (i) if there is suspicion of benignancy (masses with central scar suggesting oncocytoma, hyperattenuating lesions on unenhanced CT, or hypointense on T2 weighted-imaging MRI suggesting lipid-poor AML); (ii) in patients with surgical comorbidities [69]; (iii) in small masses (≤3 cm) due to the higher probability of benignancy [70].

### 4.2. Infiltrative Masses

Unlike the most frequently detected renal tumors, which usually present as a well-defined and encapsulated mass, infiltrative masses preserve the reniform shape and show poorly defined margins between the normal renal parenchyma and the lesion [71,72].

Infiltrative renal masses are usually primary or secondary malignant lesions, but some benign conditions such as pyelonephritis and renal sarcoidosis or post-traumatic lesions may present as infiltrative masses. The most common infiltrative renal lesions are listed in Table 3.

**Figure 9:**
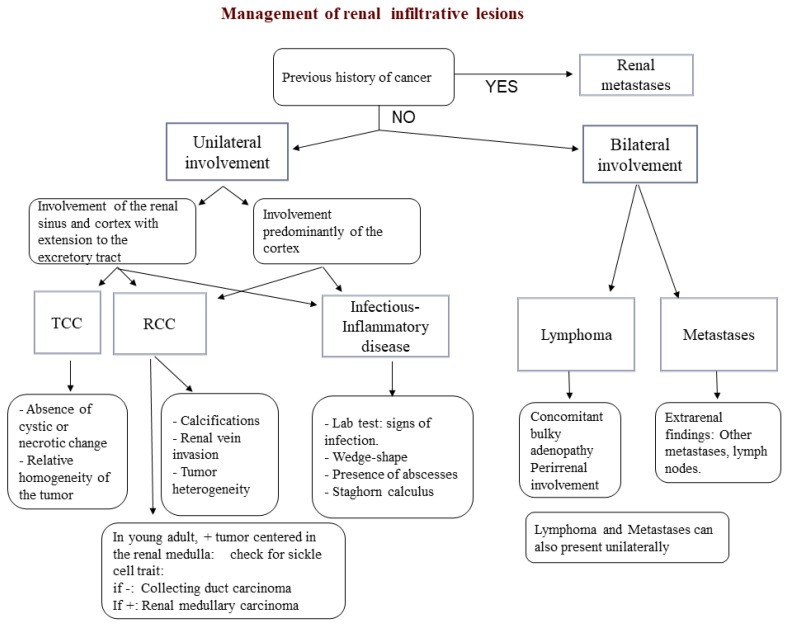
Diagnostic algorithm for the characterization of infiltrative renal masses. TCC: Transitional Cell Carcinoma.

CT and MRI usually show hypoenhancing lesions with poorly defined margins, compared with the normal renal cortex. Imaging findings may be somewhat nonspecific; thus, a combination of information from the clinical history, laboratory tests, and imaging patterns is essential to narrow the differential diagnosis. A proposed algorithm for the diagnosis of renal infiltrative masses is summarized in Figure 9.

**Figure 10:**
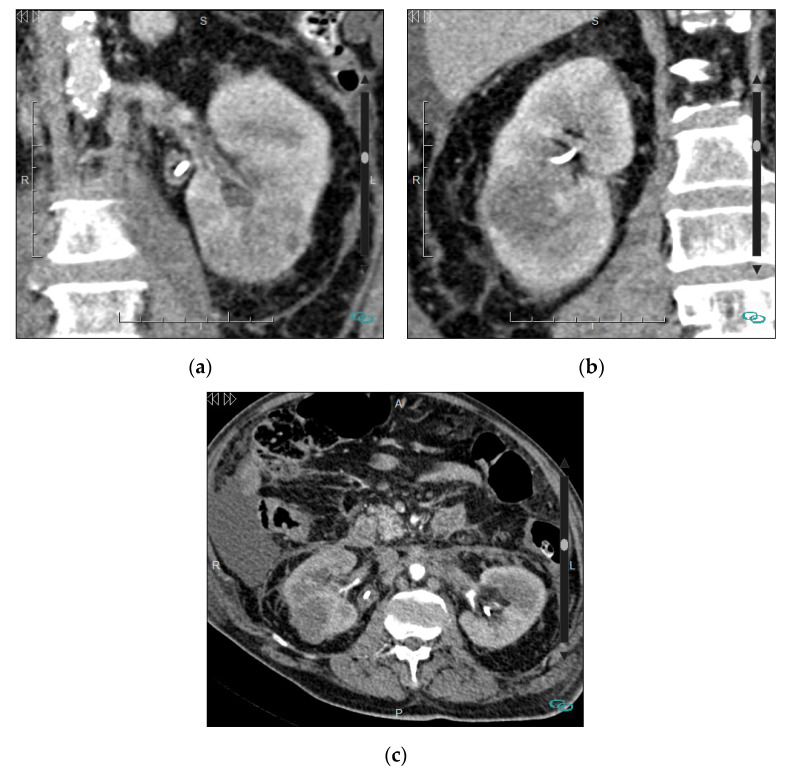
Bilateral metastases in a 65-year-old man. (**a**) Enhanced coronal CT image in portal phase of the left kidney and (**b**) right kidney show multiple cortical poorly-defined hypoenhancing lesions; (**c**) Enhanced axial CT image of the abdomen shows the multiple renal masses and also identifies the presence of stranding and nodularity of the bilateral perirenal fat, thickening of perirenal fascias, multiple retroperitoneal enlarged lymph nodes, and ascites. Diagnosis obtained by biopsy of a renal mass was metastases of high-grade carcinoma of unknown origin. Scale size: 7 cm.

In a patient with a history of cancer, the presence of an infiltrative renal mass points to metastases [72]. The most common cancers associated with renal metastasis are melanoma, lung, breast, and colorectal cancer. The presence of bilateral infiltrative masses usually corresponds to metastatic disease or lymphoma (Figure 10). In metastatic disease, both expansile and infiltrative growth patterns can be found, although the latter is uncommon. Renal metastases usually appear in patients with advanced disease; therefore, a solitary infiltrative renal lesion in a patient with a history of cancer most likely represents metastatic disease. Regarding renal lymphoma, it may be secondary to hematogenous dissemination or to contiguity of retroperitoneal lymphadenopathies. Primary isolated renal lymphoma is very rare (˂1% of all extranodal lymphomas). Renal lymphomatous involvement may present as multiple focal masses, large infiltrative lesions or diffuse bilaterally enlarged kidneys [71,73]. Ancillary findings that support the diagnosis of lymphoma are concomitant bulky lymphadenopathy and bilateral involvement [72]. Calcification is not expected in untreated lymphoma and suggests RCC.

**Figure 11:**
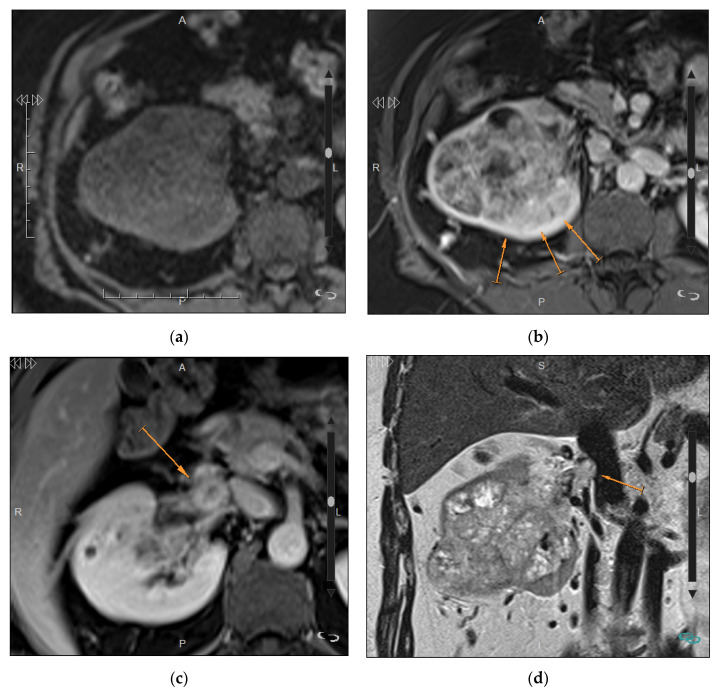
Expansive and infiltrative clear cell renal cell carcinoma in a 70-year-old man. (**a**) Axial pre-contrast T1-weighted image and (**b**) axial post-contrast T1-weighted image show a right renal mass with poorly defined margins between the mass and the adjacent renal parenchyma (arrows) with heterogeneous enhancement; (**c**) Axial postcontrast T1-weighted image also identifies a thrombus within the renal vein (arrow); (**d**) Coronal T2 weighted-image shows the heterogeneous renal mass involving the renal cortex and sinus, and confirms the thrombosis of the renal vein minimally bulging into the inferior vena cava, but without invading it (arrow). Scale size: 8 cm.

The presence of a unilateral infiltrative mass does not rule out the possibility of metastases or lymphoma. However, the most common infiltrative renal masses are RCC and transitional cell carcinoma (TCC), and they should be suggested when there is involvement of both the cortex and the renal sinus with extension to the intrarenal excretory tract. RCC may exhibit an infiltrative behavior in approximately 6% of cases [72]. The most common subtypes of renal cell carcinoma—clear cell, papillary, and chromophobe subtypes—account for over 90% of all RCCs [74], and, as such, they remain an important cause of infiltrative renal masses. Sarcomatoid variants of RCC display signs of high-grade transformation without being a distinct histological entity and are more frequently ill-defined and infiltrative lesions at imaging [72,75]. Invasion of the renal vein and/or inferior vena cava suggest RCC over other infiltrative lesions (Figure 11). There are two other types of RCCs that are usually aggressive. Renal medullary carcinoma is a very rare tumor, accounting for less than 0.5% of all RCCs [75]. It is a highly aggressive tumor, often presenting with metastases at diagnosis. It appears as an ill-defined heterogeneous hypovascular tumor, centered on the renal medulla, with associated caliectasis [76]. Features such as hemorrhage, necrosis, and regional adenopathy are common [77]. Collecting duct carcinoma is also a very rare medullary renal tumor and is often detected at an advanced stage. It usually appears as a large, central, and infiltrative tumor that is hypovascular after contrast administration [78]. Renal sinus encroachment may be observed.

On the other hand, TCC of the kidney accounts for up to 10% of neoplasms of the upper urinary tract [71]. This type of carcinoma may grow and infiltrate the adjacent renal parenchyma. CT and MRI findings are a hypoenhancing mass centered in the renal pelvis, which distorts the normal corticomedullary architecture but preserves the reniform shape. The central location, the absence of cystic or necrotic changes, and relative homogeneity of the tumor have been described as useful to differentiate intrarenal TCC from RCC [79]. However, in some cases the differential diagnosis is not possible by imaging.

The differential diagnosis of infiltrative renal masses also includes inflammatory infectious disease and the diagnosis is based on characteristic clinical symptoms and laboratory findings. Imaging techniques are used in patients who fail to respond to the appropriate therapy, for patients with recurrent infections, or for patients at significant risk of complications (elderly, diabetic, or immunocompromised). Focal nephritis foci are identified as poorly defined hypoenhancing areas of the renal parenchyma that originate from the renal papilla and extend to the renal cortex. They usually present as wedge-shaped lesions, although occasionally they can also be nodular. They are usually associated with other findings that can help in the diagnosis such as diffuse or focal renal enlargement, loss of the renal sinus fat, fat stranding, peri-nephric fluid, thickening and hyperenhancement of the collecting system, striated nephrogram, microabscesses (areas that show lack of contrast enhancement), and others [80,81].

There is currently a broad agreement in the urologic field regarding the addition of biopsy in the algorithms for the management of infiltrative renal masses [75,82]. Biopsy is advised when its result may influence clinical management; thus, it is indicated to differentiate between TCC and RCC, to differentiate between RCC, lymphoma or metastases, between malignancy versus inflammatory disease, and to obtain histology in metastasized patients.

## Figures and Tables

**Table 1:** Renal solid masses associated with a nodular growth pattern.

Nodular Renal Masses	
Renal cell carcinoma	Clear cell RCC ^1^ Papillary RCC Chromophobe RCC
Oncocytoma	
Angiomyolipoma	
Other malignant masses	Lymphoma Metastases Transitional cell carcinoma
Benign masses	Leiomyoma Adenoma
Pseudotumors	Prominent columns of Bertin, bulging of the renal contour focal renal hypertrophy

^1^ RCC = renal cell carcinoma.

**Table 2:** Imaging features of the most common nodular renal masses.

Renal Lesion	Morphologic Findings	MRI Signal Intensity	Enhancement	Diffusion
Typical AML	Macroscopic fat detection	Signal loss on Fat-saturation sequence.	Variable depending on the amount of adipose tissue, smooth muscle and blood vessels	No obvious restriction. Low signal on the ADC map due to the presence of fat.
Fat-poor AML	Hyperdense on unenhanced CT (basal CT)	Hypointense signal on T2	Variable. Usually homogeneous and prolonged	No obvious restriction.
Oncocytoma	Central scar (<50% cases)	Variable, but mainly hyper- or iso-intense.	Hyperenhancement on corticomedullary phase. Segmental enhancement inversion ^1^	No obvious restriction.
Clear cell RCC	Occasional calcifications. Occasional central scar.	May show loss of signal intensity on opposed-phased sequence (due to the presence of microscopic fat)	Hyperenhancement on corticomedullary phase Heterogeneous if haemorrhagic, cystic, or necrotic areas.	Variable restriction depending on the differentiation.
Papillary and chromophobe RCC	Occasional calcifications.	Papillary RCC may show hypointensity on T2.	Iso-hyperenhancement on nephrographic phase Homogeneous. Occasionally very scarce enhancement (papillary RCC).	Papillary RCC: Greater restriction than clear cell RCC.

^1^ Controversial finding reported in some studies and not confirmed in others; AML: Angiomyolipoma. RCC: Renal cell carcinoma. MRI = Magnetic Resonance Imaging. CT: Computed Tomography. ADC = Apparent Diffusion Coefficient.

**Table 3:** Renal solid masses associated with an infiltrative growth pattern.

**Renal cell carcinoma**	Clear cell, papillary, or chromophobe Renal medullary carcinoma Collecting duct carcinoma Sarcomatoid differentiation
**Urothelial carcinoma**	Transitional cell carcinoma Squamous cell carcinoma
**Lymphoproliferative disease**	Renal lymphoma Renal leukemia Extramedullary plasmacytoma
**Metastases**	
**Inflammatory conditions and pseudotumors**	Developmental renal pseudotumors Pyelonephritis/abscess Xanthogranulomatous pyelonephritis
